# Associations between the toe brachial index and health-related quality of life in older people

**DOI:** 10.1186/s12955-016-0451-5

**Published:** 2016-03-22

**Authors:** Jennifer Sonter, Vivienne Chuter

**Affiliations:** School of Health Sciences, Health Precinct, University of Newcastle, PO Box 127, Ourimbah, NSW 2258 Australia; School of Science and Health, Western Sydney University, Sydney, Australia; Priority Research Centre for Physical Activity and Nutrition, University of Newcastle, Ourimbah, Australia

**Keywords:** Toe brachial index, Quality of life, Peripheral arterial disease

## Abstract

**Background:**

Peripheral arterial disease is responsible for impaired health-related quality of life as a consequence of associated morbidity and mortality. The toe brachial index (TBI) provides non-invasive assessment of peripheral arterial health. Lower TBI values have been associated with foot ulceration, amputation and impaired healing, however, the relationship with health-related quality of life has not yet been investigated. The aim of this study was to explore the relationship between the TBI and health-related quality of life.

**Methods:**

This was a cross-sectional study of 100 participants aged over 50 years recruited from podiatry clinics in New South Wales, Australia. The TBI was calculated using automated equipment and quality of life was assessed using the Short Form 36 version 2 (SF-36v2). Strength of associations was determined using Pearson and Spearman correlation coefficients.

**Results:**

Lower TBI values demonstrated modest significant associations with poorer scores for the SF-36v2 domains Role Physical (*r*_s =_ 0.219, *p* > 0.05), Social Function (*r*_s_ = 0.219, *p* < 0.05) and the Physical Component Summary score (*r*_s_ = 0.203, *p* < 0.05).

**Conclusions:**

The toe brachial index demonstrates limited associations with physical and social aspects of health-related quality of life in older people.

## Background

In the lower limb, peripheral arterial disease (PAD) may remain asymptomatic or progress to such a degree that symptoms manifest as ischaemic pain, impaired wound healing and tissue loss [1]. Even in the absence of symptoms, PAD is associated with reduced physical capacity and functional decline [2, 3]. Furthermore the condition is responsible for reduced health-related quality of life (HRQoL) comparable to that of cardiovascular disease due to pain, sleep disturbance and reduced mobility [4–7]. Early diagnosis and timely risk factor modification are necessary to prevent the direct and indirect complications of PAD. Given the varied presentation and often-asymptomatic nature of the disease process, early detection relies on routine clinical screening and non-invasive tests.

The traditional office-based assessment for PAD is the ankle-brachial index (ABI), calculated as the ratio of ankle to brachial blood pressure. In the general population the ABI has been shown to have high specificity for the presence of PAD, with a low ABI considered prognostic of future cardiovascular morbidity and mortality [8, 9]. In addition, the ratio has been has been shown to correlate well with lower limb function and walking ability [10, 11] but weakly with HRQoL [12]. There is also growing evidence demonstrating limited diagnostic accuracy of this test in specific populations including those of older age and people with diabetes or significant renal disease [1, 13, 14].

The toe brachial index (TBI), calculated from the ratio of toe and brachial blood pressure is an alternative non-invasive test which has been shown to have better diagnostic accuracy for detecting PAD than the ABI in specific patient populations especially those with diabetes [15, 16]. While recent review has demonstrated there is currently insufficient literature to conclude a specific cut-off value for PAD diagnosis, the TBI was found to be useful in detecting disease regardless of the diagnostic limit used [17]. As a more distal measure of blood flow the TBI is less affected by the complications that may invalidate the ABI including medial arterial calcification [18]. Furthermore, low toe pressures have been shown to be significantly associated with higher likelihood of non-healing wounds and amputation [19]. However, there is currently limited evidence regarding the predictive capacity of the TBI for morbidity and mortality, and there has been no investigation of relationships with other measures of health including HRQoL. As a diagnostic test for PAD with greater accuracy than the ABI in at risk populations the TBI may also reflect changes in HRQoL associated with the condition and possibly to a greater extent than the ABI.

The aim of the present study was to explore the relationship between the TBI and components of HRQoL as measured using the generic Short Form 36 version 2 questionnaire (SF-36v2) in older people with and without diabetes.

## Method

### Study population

From January 2013 to February 2015, a volunteer convenience sample was recruited from patients attending routine podiatry consultation at six podiatry clinics in the Sydney and Newcastle regions of New South Wales, Australia. Inclusion criteria included those aged over 50 years with a history of diabetes or smoking and anyone aged over 65 years, consistent with recommendations for routine vascular screening [16]. Exclusion criteria included contraindications to taking toe or brachial blood pressure and inability to comply with study protocol. Seven podiatrists performed all measurements and collected participant information. Ethical approval for this study was granted by the University of Newcastle and New South Wales Health Human Research Ethics Committees. All participants including patients and clinicians gave written informed consent prior to participation in this study.

### Equipment

#### Blood pressure measurement

Toe blood pressure was measured using automated Systoe® devices (Atys Medical, Soucieu-en-Jarrest, France) consisting of an occlusion cuff, draining cuff, photoplethysmograhy probe and running unit. A pre-programed sequence of cuff inflation (to 290 mmHg) and linear deflation was used with software to detect the point of blood flow return. Brachial blood pressure was measured using MicroLife BP A100 Plus (MicroLife AG, Widnau, Switzerland) automated arm pressure cuff and unit. A TBI value was calculated for each limb by dividing the toe blood pressure by the highest of the left and right brachial systolic blood pressure. The lowest TBI from each participant was used in the analysis.

### Health-related quality of life

HRQoL was assessed using the SF-36v2Health Survey (Quality Metric). This is a reliable and valid generic health questionnaire that includes 36 questions to measure eight categories of HRQoL: physical functioning, role physical, bodily pain, general health, vitality, social functioning, role emotional, and mental health [20]. Each category is scored 1–100, with higher scores indicating better quality of life in each category. Two summary scores are generated to provide an overall approximation of mental and physical health.

### Procedures

Testing was undertaken at six clinical locations by seven podiatrists. The testing environment was maintained at 23–25 °C and participants were requested to refrain from smoking, exercising and consuming alcohol or caffeine for at least two hours prior to clinical assessment. Participants rested in a supine position for 10 min prior to blood pressure measurement at both arms and both great toes [21]. A single measurement was performed at each site. This equipment and the techniques used have previously been found to be reliable when used by different clinicians in older people [22]. SF-36v2 questionnaires were dispensed to all participants. Demographic data, smoking history (ever or never), medications and medical history including history of foot complications (ulceration and digital amputations) were recorded during the testing session. The presence of patient-reported intermittent claudication (exertional leg pain relieved by rest) and rest pain (leg pain at rest relieved by dependence) were also recorded as PAD symptoms. Participants completed the SF-36v2 questionnaire on the day of testing. The HRQoL was assessed on a 4-week recall. A researcher who was not involved in taking the measurements entered all data relating to the TBI and HRQoL.

### Statistical analyses

Quality Metric Health Outcomes ™ Scoring Software 4.5 © was used to transform SF-36v2 data. The resultant scores were then transferred to SPSS v 22 (IBM SPSS Statistics for Macintosh, Version 22, Armonk, NY, USA) for statistical analyses. Descriptive statistics were calculated and normality assessed visually using histograms and using the Shapiro-Wilk test. Pearson’s (*r*) and Spearman’s rho correlation coefficient (*r*_s_) was used to assess the presence and strength of associations between the TBI and HRQoL [23], and were interpreted according to Cohen [24] (<0.1 insubstantial, 0.1–0.3 small, 0.3–0.5 moderate, > 0.5 large). Significance was set at an alpha level of 0.05. T-tests were performed to establish if a difference exists between HRQoL between participants with and without diabetes, with and without a history of smoking, and males and females.

## Results

### Participants

One hundred participants underwent blood pressure measurements and completed the SF-36v2 questionnaire. The mean age of participants was 70 ± 9.55 years (range 50–92 years), 53 % were male and 55 % had diabetes. The mean TBI value was 0.79 ± 0.21. Twenty-eight percent of participants were found to have PAD using a cut-off value of 0.70. Participant characteristics are presented in Table 1.

**Table 1:** Participant characteristics

	Diabetes	No Diabetes	Total
n	55	45	100
Males	32 (58 %)	21 (53 %)	53 (53 %)
Age (years)	68 ± 9*	72 ± 10	70 ± 10
Body mass index (kg/m^2^)	32.11 ± 7.87	29.54 ± 6.45	30.95 ± 7.34
Toe brachial index	0.76 ± 0.22	0.82 ± 0.19	0.79 ± 0.21
Smokers (ever)	18 (33 %)	16 (36 %)	34 (34 %)
Hypertension	30 (55 %)	22 (49 %)	52 (52 %)
Dyslipidaemia	18 (33 %)	15 (33 %)	33 (33 %)
History ulcer or amputation	3 (5 %)	1 (2 %)	4 (4 %)
Intermittent claudication or rest pain	3 (6 %)	2 (4 %)	5 (5 %)

Mean ± standard deviation

*Significant difference between groups (*p* < 0.05)

### Health-related quality of life

The SF-36v2 questionnaire was complete for all participants. Visual impairment required an investigator read the questionnaire aloud to one participant. Median scores for each HRQoL domain are presented in Table 2 and mean scores in Table 3.

**Table 2:** Median SF-36v2 scores

	Median (25th, 75th centiles)
Physical function	70 (45–85)
Role physical	72 (38–94)
Bodily pain	62 (41–84)
Social function	81 (50–100)
Role emotional	83 (52–100)
Mental health	80 (65–90)
Physical component summary	46 (37–52)
Mental component summary	53 (43–58)

**Table 3:** Mean SF-36v2 scores

	Mean (Standard deviation)
General health	59 (22)
Vitality	53 (20)

### Correlation analysis

Spearman and Pearson correlations between the TBI and SF-36v2 domains are shown in Tables 4 and 5, respectively. Weak yet significant positive correlations were found between the TBI and HRQoL for the domains role physical (*r*_s_ = 0.219, *p* = 0.029), social functioning (*r*_s_ = 0.219, *p* = 0.028), and the physical component summary (*r*_s_ = 0.203, *p* = 0.042). Sub-group analysis determined no significant difference in HRQoL domains in participants with and without diabetes, of male and female gender, or those who smoked and those who never smoked (Table 6).

**Table 4:** Spearman correlations between SF-36v2 domains and the TBI, PAD symptoms, and complications

	Toe brachial index		PAD symptoms		Foot complications	
SF-36 v2 Domain	*r*_s_	*p*	*r*_s_	*p*	*r*_s_	*p*
Physical function	0.112	0.267	0.030	0.765	−0.126	0.212
Role physical	0.219	0.029*	−0.206	0.039*	−0.222	0.027*
Body pain	0.140	0.165	0.093	0.355	0.004	0.972
Social Function	0.219	0.028*	0.052	0.608	0.094	0.355
Role Emotional	0.163	0.104	−0.037	0.715	0.015	0.885
Mental health	−0.004	0.969	−0.018	0.856	0.030	0.766
Physical component summary	0.203	0.042*	0.019	0.851	−0.160	0.112
Mental component summary	0.088	0.386	0.012	0.906	0.122	0.227

*PAD* peripheral arterial disease, *r*_*s*_ Spearman’s correlation coefficient. *significance level < 0.05

**Table 5:** Pearson correlations between SF-36v2 domains and the TBI, PAD symptoms and foot complications

	Toe brachial index		PAD symptoms		Foot complications	
SF-36 v2 Domain	*r*	*p*	*r*	*p*	*r*	*p*
General health	0.129	0.202	0.068	0.499	−0.038	0.709
Vitality	0.135	0.179	0.032	0.755	−0.064	0.526

*PAD* peripheral arterial disease, *r* Pearson’s correlation coefficient

**Table 6:** T-test for domain differences between sub-groups

	Diabetes vs no diabetes	Smokers vs non-smokers	Males vs females
Physical function	0.571	0.620	0.231
Role physical	0.762	0.343	0.342
Bodily pain	0.864	0.234	0.274
General health	0.561	0.460	0.092
Vitality	0.690	0.124	0.152
Social function	0.250	0.341	0.213
Role emotional	0.232	0.414	0.111
Mental health	0.261	0.242	0.412

## Discussion

This study of 100 participants is the first investigation of the relationship between the TBI and HRQoL. The mean TBI value in this population was 0.79 (±0.21), a value above the threshold for the diagnosis of PAD that suggests the population as a whole was generally without severe disease of the peripheral vasculature. Despite this, people with lower TBI values were found to have reduced HRQoL for the SF-36v2 domains Social Function, Role Physical, and the Physical Component Summary.

People with lower TBI values had greater and more frequent interference with normal social activities due to physical and emotional problems as indicated by poorer scores for the SF-36v2 Social Function domain (*r*_s_ = 0.219, *p* = 0.028) [25]. Reduced social functioning has previously been shown in people with intermittent claudication symptoms from significant PAD, which is proposed to lead to feelings of inadequacy and may result in social isolation [6, 26]. In this present study PAD symptoms were not found to correlate significantly with the Social Function domain. However this is likely to have been confounded by the very small number of participants recruited who reported painful PAD symptoms. Had the participant cohort had a lower mean TBI, indicative of more frequent and more severe pathology it is possible a similar relationship between PAD symptoms and reduced Social Function would have been demonstrated. Furthermore, the TBI is reflective of lower limb vascular health generally and may be affected by disease other than PAD including microvascular diseases such as peripheral neuropathy. It is possible that investigation of a study population with a lower mean TBI would demonstrate associations with other or atypical symptoms responsible for physical or emotional limitations of social function, including neuropathy and microvascular disease.

This study also demonstrated a significant relationship between lower TBI values and the SF-36v2 Role Physical domain (*r*_s_ = 0.219, *p* = 0.029). This scale reflects problems with work or other daily activities that result from physical health and our results indicate people with lower TBI values are more likely to be limited in the type and amount of work they perform [25]. This domain also demonstrated significant inverse correlations with foot complications (*r*_s_ = −0.222) and PAD symptoms (*r*_s_ = −0.206). The TBI has recently demonstrated significant correlations with PAD symptoms and foot complications in older people and may additionally reflect physical limitations associated with these [Unpublished observations Sonter, J. and Chuter V].

While the painful symptoms of PAD are known to limit activity and to correlate significantly with reduced Role Physical HRQoL, many people with PAD have atypical symptoms or are asymptomatic. Indeed previous studies have reported approximately 30–45 % of people with PAD will experience atypical symptoms [27–30]. Even in these cases, the level of impairment may greater than for those without PAD [3, 4, 31]. In the present study the population was generally without PAD and yet a modest significant association between the TBI and lower Physical Component Summary score was found (*r*_s_ = 0.203, *p* < 0.05). The median score for the Physical Component Summary was the lowest of all SF-36v2 domains assessed in this population [46 (37–52)]. Low scores on this scale encompass limitations in self-care, physical, social and role activities, body pain, tiredness, and a poor health rating [32]. Given the similarly lower TBI values associated with reductions in both Social Function and Role Physical, a significant association with this composite score is unsurprising. In a study of people with peripheral vascular disease, the ABI also demonstrated a small correlation with the SF-36 Physical Component Summary score [12]. As both the ABI and TBI are used to assess the peripheral vasculature, the similar results are likely due to both reflecting PAD-associated physical impairment. More specific physical impairments including reduced walking speed, activity level and standing balance have been associated with the ABI [10]. As a similar measure with potentially greater utility in at-risk populations, the TBI could also reflect reductions in these physical functions and activities. In the present study 28 % of participants were found to have a TBI of less than 0.70, a suggested diagnostic threshold for PAD. As only 5 % of participants reported traditional symptoms the correlations found may underestimate the relationship between the TBI and HRQoL. Further investigation of the functional limitations occurring at lower TBI values would provide a clearer understanding of the specific lower limb impairments associated with the index and the effect of these on HRQoL.

### Limitations

The results of this study should be interpreted in the context of its limitations. It is important to acknowledge that many factors influence HRQoL and causality was not investigated in this study. This was a cross-sectional study and as such only correlations have been reported. Given the older age of participants in the present study and generic SF-36v2 tool used it is likely the results are affected by other factors unrelated to PAD or the TBI. The overall mean TBI indicated most of the population was without significant pathology, therefore it is likely that the correlations would be stronger in a more symptomatic population. However, this population is reflective of community-based populations attending podiatry services that require vascular screening. The TBI values obtained in this study may have been falsely low due to normal day to day variation in blood pressure and temperature however this methodology has been utilised previously with good diagnostic efficacy [33]. Lastly, a generic HRQoL was chosen for the present exploratory investigation, however, future studies may benefit from the inclusion of disease-specific and functional assessment tools.

## Conclusions

The study provides the first evidence of a relationship between the TBI and HRQoL. In an older population lower TBI values were associated with role limitations due to physical health, with impaired social function. The results presented here indicate the TBI may be useful beyond its role as a non-invasive assessment of peripheral arterial health. In addition it may indicate impaired aspects of HRQoL that are not reflected by signs and symptoms of PAD. Future investigations to determine the association between the TBI and HRQoL using PAD-specific HRQoL tools are now warranted. Additional information is required to determine whether the TBI can be used clinically to identify patients at risk of impaired HRQoL.
